# Cu-Sn Aerogels for Electrochemical CO_2_ Reduction with High CO Selectivity

**DOI:** 10.3390/molecules28031033

**Published:** 2023-01-19

**Authors:** Yexin Pan, Muchen Wu, Ziran Ye, Haibin Tang, Zhanglian Hong, Mingjia Zhi

**Affiliations:** 1 State Key Laboratory of Silicon Material, School of Materials Science and Engineering, Zhejiang University, 38 Zheda Road, Hangzhou 310027, China; 2 Department of Applied Physics, Zhejiang University of Technology, Hangzhou 310014, China; 3 Key Laboratory of Materials Physics, and Anhui Key Laboratory of Nanomaterials and Nanotechnology, Hefei Institute of Solid State Physics, Chinese Academy of Sciences, Hefei 230031, China; 4 Institute for Composites Science Innovation (InCSI), Zhejiang University, 38 Zheda Road, Hangzhou 310027, China

**Keywords:** alloy, aerogel, CO_2_ reduction, CO selectivity

## Abstract

This work reports the synthesis of Cu_x_Sn_y_ alloy aerogels for electrochemical CO_2_ reduction catalysts. An in situ reduction and the subsequent freeze-drying process can successfully give CnxSny aerogels with tuneable Sn contents, and such aerogels are composed of three-dimensional architectures made from inter-connected fine nanoparticles with pores as the channels. Density functional theory (DFT) calculations show that the introduction of Sn in Cu aerogels inhibits H_2_ evolution reaction (HER) activity, while the accelerated CO desorption on the catalyst surface is found at the same time. The porous structure of aerogel also favors exposing more active sites. Counting these together, with the optimized composition of Cu_95_Sn_5_ aerogel, the high selectivity of CO can be achieved with a faradaic efficiency of over 90% in a wide potential range (−0.7 V to −1.0 V vs. RHE).

## 1. Introduction

Electrochemical CO_2_ reduction reaction (CO_2_RR) can convert CO_2_ to useful fuels and chemicals, and the efficiency of such a reaction highly depends on the electrocatalysts [1,2,3,4]. Despite the different types of new catalysts reported recently [5,6,7,8,9], copper (Cu) still holds its advantages of low cost, being earth-abundant, and the capability to convert CO_2_ to a series of chemicals, including CO, hydrocarbons, and other value-added liquid alcohol and acid [10]. Further improving the selectivity to a specific product, minimizing the overpotential, and suppressing the main competing H_2_ evolution reaction (HER) are the key to developing Cu-based electrocatalysts [11], and this has been accomplished by several methods, such as adopting oxide-derived Cu [12], building Cu-oxide heterojuctions [13,14,15], constructing Cu complexes [16], and alloying Cu with other elements [17].

Alloying or constructing bimetallic compounds can alter the local geometric and/or electronic structures and bring new active metal sites [18,19,20]. Both theoretical calculations and experimental works have shown that the selectivity towards CO can be greatly improved in a Cu alloy or bimetallic electrocatalyst, due to the change in binding strength between the reaction intermediates and the catalyst surface. In the literature, CuIn [21], CuAg [22], CuPd [23], and CuSn alloys [24,25] have been extensively studied. Among the possible elements, Tin (Sn) is one of the effective candidates due to its unique activities and low cost [26]. Porous Cu-Sn electrodes are favorable to producing formate during CO_2_RR [27]. A similar phenomenon has been observed in electrodeposited Cu-Sn alloy on carbon fibers [25]. In CuSn systems, previous studies showed that controlling the geometry or the composition can improve the catalytic performance for CO_2_ reduction. For example, monodisperse Cu core/SnO_2_ shell nanoparticles prepared by the seed-mediated method have shown a faradaic efficiency (FE) of CO reaching 93% at −0.7 V vs. RHE [28]. Takanabe et al. reported a Cu-Sn bimetallic catalyst by electrodeposition Sn species on the surface of oxide-derived Cu, which can selectively produce CO (CO faradaic efficiency > 90%) over a wide potential range [29]. However, such electrodes often suffer from a low total current density compared with their pristine Cu counterparts.

Further optimizing such electrodes could be realized by adopting porous structures to offer more active sites while the selectivity may be retained. Aerogels that have intrinsic features, such as large surface area, high porosity, and abundant active sites, would fulfill such requirements, especially for catalytic reactions [30,31,32,33,34]. Eychmüller’s pioneer work has proven the feasibility of preparing various alloy hydrogels in aqueous solution, and the yielded aerogels have often shown superior activity compared to their nanoparticle analogs because of the special properties of nanomaterials which are incorporated and enlarged on a macroscopic scale [35]. Apart from the well-studied noble metal alloy aerogels, Cu-based aerogel has also attracted a great deal of interest. A series of MCu (M = Pd, Pt, and Au) alloy aerogels can serve as unsupported electrocatalysts, providing enhanced oxygen reduction reaction catalytic activities and high stabilities [36]. Therefore, suitable Cu-based aerogels could be a promising candidate to drive CO_2_RR.

Inspired by the above discussion, in this study, we present the work to employ Cu_x_Sn_y_ alloy aerogels as electrocatalysts for electrochemical CO_2_RR. By using the well-established simple co-reduction and gelation process, Cu_x_Sn_y_ hydrogel can readily form. The synthesis relies on the rapid reduction of Cu and Sn cations in a liquid medium and the populated nanoparticles form a porous gel structure. After freeze-drying, porous Cu_x_Sn_y_ alloy aerogels with large surface area and pore volume are obtained. Such an aerogel preserves the well-known HER suppression effect brought by the Sn additive and the open structure for exposing electrochemical active sites. As a result, a robust and highly efficient Cu_x_Sn_y_ electrocatalyst that can selectively reduce CO_2_ to CO is developed. Potentially with the newly emerged new preparation methods for fine metal nanoparticles, such as microfluid assistant synthesis [37,38], it would be feasible to explore more possible applications of such metal aerogels.

## 2. Experimental

### 2.1. Synthesis of Cu_x_Sn_y_ Aerogels

The preparation of the aerogel includes the preparation of the hydrogel and the freeze-drying of the hydrogel to the aerogel. The Cu_x_Sn_y_ hydrogels were synthesized by simply mixing CuCl_2_ (analytical grade, Sinopharm chemical reagent) and SnCl_4_ (analytical grade, Sinopharm chemical reagent) with NaBH_4_ (analytical grade, Sinopharm chemical reagent) in an aqueous solution, which has been reported for preparing various noble metal hydrogels [39,40]. Taking the preparation of the Cu_95_Sn_5_ hydrogel as an example, 0.756 mmol CuCl_2_·2H_2_O and 0.084 mmol SnCl_4_·5H_2_O were completely dissolved in 70 mL H_2_O and stirred at 60 °C for 10 min, and a green solution was obtained. After that, 7 mL of 2 M NaBH_4_ aqueous solution was quickly injected into the above solution under stirring. The color of the mixture turned black immediately. The obtained suspension was further kept at 60 °C for 3 h and a black monolithic hydrogel gradually emerged at the bottom of the container. The hydrogel was then collected with caution and washed with H_2_O several times to remove the soluble impurities and by-products. The hydrogel monolithic was loaded into a freeze dryer (FD-1A-80, Boyikang, Beijing, China) at 195 K and then vacuumed for 48 h to give Cu_95_Sn_5_ aerogel. Furthermore, the molar ratio of CuCl_2_ and SnCl_4_ can be adjusted for the preparation of other Cu_x_Sn_y_ aerogels with different compositions. By the identical protocol, Cu_97_Sn_3_, Cu_95_Sn_5_, and Cu_90_Sn_10_ aerogels were prepared (the detail can be seen in the supporting information Table S1). Pristine Cu and Sn aerogels were also prepared as control samples.

### 2.2. Materials Characterization

X-ray diffraction (XRD) patterns of the samples were collected on SHIMADZU XRD-6000. The morphology was observed by using HITACHI S-4800 scanning electron microscopy (SEM) and JEOL-2100F transmission electron microscopy (TEM). X-ray photoelectron spectroscopy (XPS) data were obtained on Thermo Scientific K-Alpha using monochromatic Al Kα radiation. The nitrogen adsorption/desorption isotherms of the samples were performed using Micromeritics ASAP2460. The exact content of the chemical element was obtained with an inductively coupled plasma optical emission spectrometer (ICP-OES, Agilent 720ES). Temperature-programmed desorption mass spectrometry (TPD-MS) of CO on the samples was performed on AutoChem1 II 2920.

### 2.3. Electrochemical Measurements

In a typical experiment, 5 mg of aerogel was dispersed in a solvent of 400 μL ethanol, 100 μL deionized water, and 20 μL 5 wt% Nafion solution (Sigma-Aldrich) to form the ink. Then, 100 μL ink was dropped on carbon paper (1 cm × 1 cm) and dried in a vacuum oven as the working electrode. Ag/AgCl and Pt foil were used as the reference electrode and counter electrode, respectively. A 0.1 M KHCO_3_ solution was used as the electrolyte and purged with CO_2_ gas (20 mL min^−1^) to ensure sufficient CO_2_ transport to the electrode surface during the testing.

The electrochemical CO_2_ reduction was conducted on an IVIUM VERTEX electrochemical workstation using a typical gas-tight H-type cell, separated with a Nafion 117 proton exchange membrane. The electrolyte was constantly stirred during the testing. The linear sweep voltammetry (LSV) measurement was performed from 0 V to −1.1 V vs. RHE at 10 mV s^−1^. Potentiostatic electrochemical reduction of CO_2_ was investigated at different potentials. The gas products were quantified with a gas chromatograph (GC, Fuly GC9720Plus) equipped with a thermal conductivity detector (TCD) and a flame ionization detector (FID). The GC was calibrated by the standard gas before each measurement. The detailed calculation of the Faradaic efficiencies of gaseous products can be found in the supporting information.

## 3. Results and Discussion

**Figure 1:**
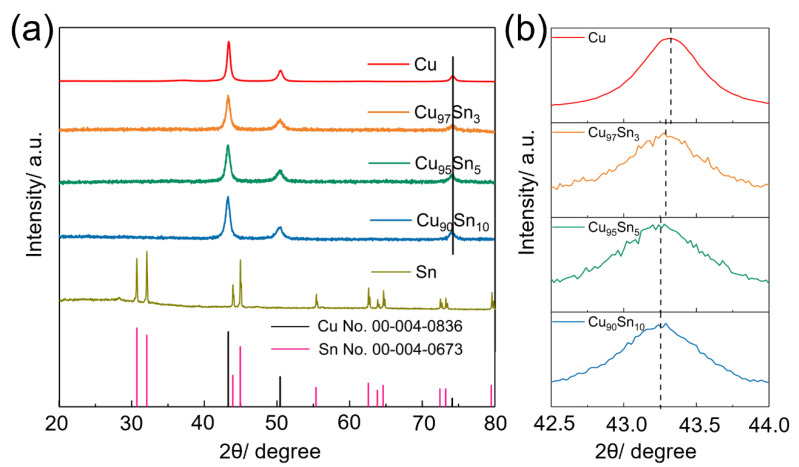
(**a**) XRD patterns of Cu, Cu_97_Sn_3_, Cu_95_Sn_5_, Cu_90_Sn_10_, and Sn aerogels, and (**b**) the closer view of (111) peak.

Pristine Cu, Sn, and Cu_x_Sn_y_ alloy aerogels (including Cu_97_Sn_3_, Cu_95_Sn_5_, and Cu_90_Sn_10_) are obtained via the in situ reduction of the metal cations and freeze-drying. The detailed chemicals can be found in Table S1. Figure 1a gives the XRD (X-ray diffraction) patterns of the corresponding samples. The diffraction peaks of the pristine Cu and Sn samples can well match with the standard Cu (JCPDS#00-004-0836, cubic) and Sn (JCPDS#00-004-0673, tetragonal) crystals. This proves that NaBH_4_ can effectively reduce the high valence Cu and Sn cations to zero valence and form crystalized metal species. Cu_x_Sn_y_ aerogels have similar diffraction peaks to that of pristine Cu aerogel despite their different compositions, and no other notable oxide impurities or peaks to Sn can be found. The observed diffraction peaks can be assigned to (111), (200), and (220) planes of Cu. Similar patterns have been found in Cu-Sn alloy samples in the literature [41,42], suggesting that the CnSn alloy nanowires and Cu-Sn foam have an identical set of patterns to the standard Cu. A closer view of the (111) peak of Cu_x_Sn_y_ series samples in Figure 1b reveals that the peak shifts to a lower angle, which is a sign of the expanded crystalline lattice. Considering that the radius of Sn atoms (140 pm) is larger than that of Cu (128 pm), the crystalline phase of the Cu_x_Sn_y_ aerogel can be identified as a Cu-Sn alloy, and Sn atoms are implanted into the Cu crystalline lattice. According to the Bragg equation, the (111) plane spacing of the Cu, Cu_97_Sn_3_, Cu_95_Sn_5_, and Cu_90_Sn_10_ samples was calculated as 0.2087 nm, 0.2089 nm, 0.2090 nm, and 0.2090 nm, respectively, which was consistent with the peak shift. The broad diffraction feature also confirms the aerogels are composed of fine nanoparticles. Based on Sherrer’s equation, the calculated crystalline sizes in Cu, Cu_97_Sn_3_, Cu_95_Sn_5_, and Cu_90_Sn_10_ aerogels are ~17.3 nm, 13.1 nm, 11.8 nm, and 11.9 nm, respectively.

The chemical states of the elements in the aerogels were further characterized by XPS (X-ray photoelectron spectroscopy) measurements. Figures S1 and S2 give detailed scans of Cu 2p and Sn 3d core levels of the aerogels. Generally, the Cu 2p of the aerogels can be well fitted by two pairs of peaks, which correspond to Cu^+^/Cu^0^ (2p3/2 at 932.63 eV and 2p1/2 at 952.43 eV, separation 19.8 eV) and Cu^2+^ (2p3/2 at 934.83 eV and 2p1/2 at 954.83 eV, separation 20.0 eV) [28,43]. The component of Cu^+^/Cu^0^ is dominated in the spectra and matched with XRD. The Sn 3d spectra show the Sn 3d5/2 maximum at 486.4 eV, which is related to the presence of Sn (IV), together with a small shoulder at 485.9 eV due to Sn (II) [24,28,42]. The component of Cu^2+^ and Sn (IV) and Sn (II) may mainly result from the surface oxidation of the aerogels upon exposure to air [42]. The overall Cu:Sn composition determined by inductively coupled plasma atomic emission spectrometry (ICP-AES) is ~97:3, 95:5, and 90:10 for Cu_97_Sn_3_, Cu_95_Sn_5_, and Cu_90_Sn_10_ aerogels, respectively, as shown in the supporting information Table S1. It should be pointed out that the Sn content in the final aerogel is always lower than that in the precursors, which may be due to the different hydrolysis rates of Cu and Sn cations.

**Figure 2:**
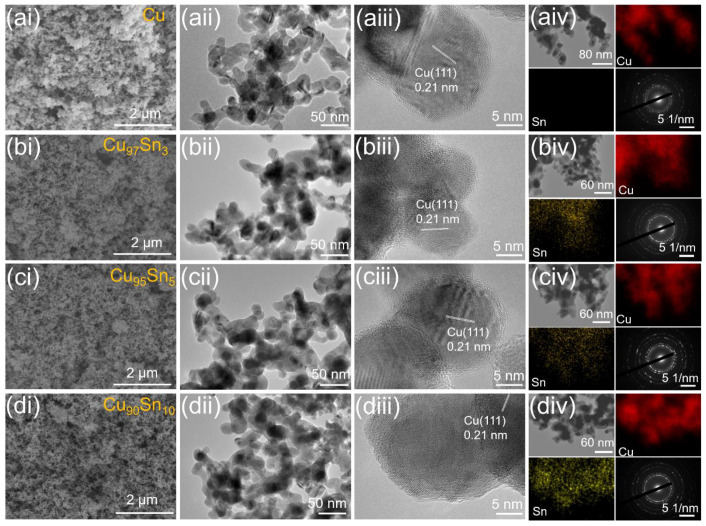
SEM, TEM, HRTEM, EDX mapping images and SAED patterns of Cu (**ai**–**aiv**), Cu_97_Sn_3_ (**bi**–**biv**), Cu_95_Sn_5_ (**ci**–**civ**), and Cu_90_Sn_10_ (**di**–**div**) aerogels.

The freeze-drying process of the hydrogel ensures the porous structure of the aerogels. The morphology of the Cu and Cu_x_Sn_y_ aerogels are characterized by SEM (scanning electron microscope) and TEM (transmission electron microscope), and the results are displayed in Figure 2. The appearance of the pristine Cu aerogel (Figure 2ai) shows a typical 3D porous structure composed of fine nanoparticles, which is a characteristic morphology for aerogels and is similar to that of the Pt_x_Pd_y_ aerogel [44] and Pd aerogel [35] reported in the literature. The TEM image in Figure 2aii further revealed the microstructure of the interconnected nanoparticles with plenty of voids between them. The average size of the nanoparticles is around 20 nm, which is close to the value calculated from XRD. HRTEM image indicates that an individual nanoparticle is highly crystallized, and a lattice fringe of 0.21 nm can be seen, which corresponds to the (111) interplane distance of Cu (Figure 2aiii). This point is further confirmed by the selected area electron diffraction (SAED) pattern in Figure 2aiv since clear diffraction dots are presented. With the incorporation of Sn, there is no obvious change in the microstructure as shown in Figure 2bi–biv,ci–civ,di–div, and a porous nanoparticle skeleton is always seen. This indicates that the reduction and gelation processes were not significantly affected by the composition of the precursor. The EDX mappings of Cu_x_Sn_y_ aerogels show a uniform distribution of Sn and Cu elements within the aerogel framework, further indicating an alloy has formed.

The surface area and the porosity of the aerogels samples are obtained by N_2_ physisorption experiments at 77 K. Figure S3 displays the isothermal adsorption/desorption curves, and the inset is the pore size distribution plots which are assessed by Barrett–Joyner–Halenda (BJH) method. The surface areas estimated from Brunauer–Emmett–Teller (BET) theory are 23.1 m^2^ g^−1^, 29.5 m^2^ g^−1^, 32.2 m^2^ g^−1^, and 29.7 m^2^ g^−1^ for the Cu, Cu_97_Sn_3_, Cu_95_Sn_5_, and Cu_90_Sn_10_ aerogels, respectively. The values are within the range of the reported MCu (M= Pd, Pt, and Au) alloy aerogels prepared by a similar method [36]. All the aerogels show a wide range of pores in the mesopores ranges. At high relative pressure (P/P0), no plateau appears in the adsorption isotherm, which implies the presence of macropores at the same time [35]. From SEM and TEM images, it is also clear that there are mesopores and macropores in the aerogels. The pore volumes are 0.11 cm^3^ g^−1^, 0.20 cm^3^ g^−1^, 0.21 cm^3^ g^−1^, and 0.22 cm^3^ g^−1^ for the Cu, Cu_97_Sn_3_, Cu_95_Sn_5_, and Cu_90_Sn_10_ aerogels, respectively. Interestingly, the pore size distributions of the Cu aerogel and Cu_x_Sn_y_ aerogels are different. The majority of the pores in the Cu aerogel are below 10 nm, while there are larger pores (proved by the elevated tails in the pore size distribution plots above 10 nm) presented in Cn_x_Sn_y_ aerogels. Potentially, these pores can act as the microchannels for reactant diffusion and product releasing in the electrode.

**Figure 3:**
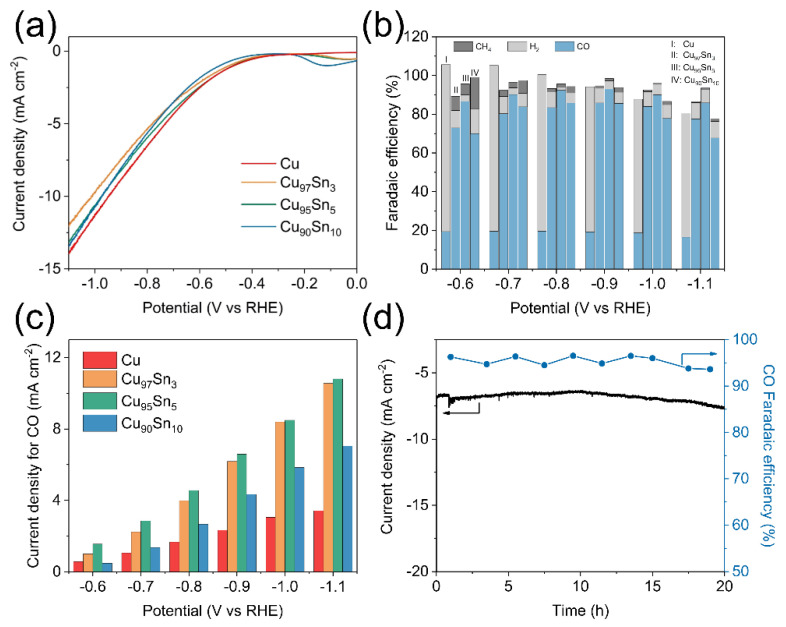
Electrochemical CO_2_ reductions in 0.1 M KHCO_3_. (**a**) LSV curves for Cu, Cu_97_Sn_3_, Cu_95_Sn_5_, Cu_90_Sn_10_ aerogels. (**b**) Faradaic efficiency for Cu, Cu_97_Sn_3_, Cu_95_Sn_5_, Cu_90_Sn_10_ aerogels. (**c**) CO partial current density for Cu, Cu_97_Sn_3_, Cu_95_Sn_5_, Cu_90_Sn_10_ aerogels. (**d**) Current density and faradaic efficiency of long-term electrolysis over Cu_95_Sn_5_ aerogel electrodes at −0.9 V vs. RHE.

The electrocatalytic activity of the Cu and Cu_x_Sn_y_ aerogels is investigated by controlled-potential electrolysis in the CO_2_-saturated 0.1 M KHCO_3_ electrolyte. The linear sweep voltammetry (LSV) curves of Cu and Cu_x_Sn_y_ aerogels are shown in Figure 3a. The applied potential was swept at a rate of 10 mV s^−1^. A first glance at the curves indicates that the total current densities at different potentials did not show a significant difference among the samples. The curves all have a small current density from 0.0 to −0.5 V vs. RHE, and it increases dramatically afterward, indicating an electrochemical reduction reaction occurring. The current densities above −0.6 V vs. RHE almost overlapped in Cu and Cu_x_Sn_y_ series aerogels, while a small difference is found when the potential is swept beyond −0.6 V. The final current densities at −1.1 V vs. RHE are −14.0, −12.0, −13.1, and −13.4 mA cm^−2^ for the Cu, Cu_97_Sn_3_, Cu_95_Sn_5_, and Cu_90_Sn_10_ aerogel samples, respectively. This indicates that alloying Sn into Cu aerogel did not significantly deteriorate the electron-transfer rates of Cu. This point is different from the literature, in which it is found that adding Sn to Cu would often lower the overall current density. For instance, the current density of a Cu-Sn bimetallic catalyst reduces significantly compared to that of the pristine Cu, and similar behavior was observed in the CuSn nanowires catalyst [29,43]. The products of different aerogel electrodes show huge differences. Figure 3b gives the Faradaic efficiencies (FE) of the pristine Cu and CuxSny aerogels electrodes from −0.6 V to −1.1 V vs. RHE. For pristine Cu aerogel electrodes, it always exhibits a low FE of CO and a much higher FE of H_2_. Within the potential range, the FE of CO remains at a constant value of about 20%, while the remaining product is dominated by H_2_. The above results are consistent with the literature that HER is the main competing reaction to CO_2_RR on the Cu electrode, and a high FE of H_2_ (>80%) is usually seen [45]. In Cu_x_Sn_y_ series aerogels, even a small amount of Sn can significantly elevate the FE of CO_2_RR, and CO became the main product. The FE of CO for the Cu_97_Sn_3_ aerogel was 80% at −0.7 V vs. RHE, while this value can reach 90% in the Cu_95_Sn_5_ aerogel, and such FE of CO can maintain above 90% in a broad potential range from −0.7 V to −1.0 V vs. RHE. The highest CO FE was 93% at −0.9 V vs. RHE in the Cu_95_Sn_5_ aerogel. Further increasing Sn content in the aerogel or sweeping the potential to a more negative range causes the decrease in CO FE. For instance, the FE of CO in the Cu_90_Sn_10_ aerogel was 86% at −0.9 V vs. RHE. At −1.1 V vs. RHE, FE of CO in all Cu_x_Sn_y_ aerogels dropped to below 90% (Cu_97_Sn_3_: 78%; Cu_95_Sn_5_: 86%, and Cu_90_Sn_10_: 68%). Additionally, a trace amount of CH_4_ is detected as the by-product. The sum of the faradaic efficiency of these gas-phase products at −0.9 V vs. RHE is close to 100%, indicating no other main species are produced. At a more negative potential, the total faradaic efficiency of the gas phase product is below 90%, indicating the possible liquid products. The partial current density of H_2_ and CO can be calculated based on the chronoamperometry curves and the results are shown in Figure 3c and Figure S4. In line with the trend of Faraday efficiency, with Sn additives, the H_2_ partial current densities dropped dramatically, and the CO partial current densities raised notably compared with those in the pristine Cu. The H_2_ partial current density for the Cu aerogel is 9.07 mA cm^−2^ at −0.9 V vs. RHE, and it decreased to 0.54 mA cm^−2^ in the Cu_97_Sn_3_ aerogel. As for the Cu_95_Sn_5_ and Cu_90_Sn_10_ aerogels, the H_2_ current density is similar and lowers to ~0.29 mA cm^−2^. The CO partial current density for the pristine Cu aerogel is 2.33 mA cm^−2^ at −0.9 V vs. RHE, and it increases almost 3-fold in the Cu_97_Sn_3_ aerogel (6.18 mA cm^−2^) and further to 6.58 mA cm^−2^ in the Cu_95_Sn_5_ aerogel. However, when the Sn content increases to Cu_90_Sn_10_, the current density of CO decreases to 4.33 mA cm^−2^. Therefore, among the above materials, the Cu_95_Sn_5_ aerogel shows the lowest HER activity and the highest CO production rate, which results in it having the highest selectivity for CO. A comparison of CO partial current of the Cu_95_Sn_5_ aerogel and other Cu-Sn bimetallic [29,42], Cu-In bimetallic [21], and Cu-Ag [46] bimetallic catalysts can be found in Table S2, which highlights its high activity. The durability of the Cu_95_Sn_5_ aerogel was further verified by constant potential electrolysis at −0.9 V vs. RHE for 20 h and the I-t curve is shown in Figure 3d. The current density did not show a notable decay during the long-term stability testing, and the FE of CO remained above 90% during the long-term testing. The TEM images (Figure S5) before and after electrolysis show that the microstructure of the Cu_95_Sn_5_ aerogel is still composed of nanoparticles, although the porosity was reduced in the tested sample.

The above results confirm that the Cu_95_Sn_5_ alloy aerogel possesses high activity and high selectivity to CO. The activity is featured by the higher CO partial current density while the selectivity is highlighted by the high CO FE. The former can be partially ascribed to the highly porous unsupported network, which may favor the exposure of electrochemically active sites and a high reactant flux. Such a strategy has been well-proven in other bimetallic aerogels as an efficient electrocatalyst [39,47]. The latter has been reported in the previous literature since high selectivity to CO or HCOOH is found in bimetallic Cu-based nanostructures, such as Cu-Sn [24,29,41], Cu-Pd [23], and Cu-In [21]. The CO partial current at −0.9V vs. RHE (6.58 mA ccm^−2^) and the Faradaic efficiency are comparable to this work. A straightforward explanation for such a phenomenon is the suppression of the HER side reaction. For instance, Sarfraz’s work reported that depositing an additional layer of Sn on the Cu electrode can significantly increase CO FE from 63% to more than 90% at −0.6 V vs. RHE [29]. However, a great decrease in the total current (−1.0 mA cm^−2^ for Cu-Sn and −2.1 mA cm^−2^ for Cu) was observed at the same time, which indicates that the overall activity of the electrode decreased. Such increased CO FE can then be understood by the combination of the decreased total current (as a denominator) and the barely unchanged CO current (as a numerator). Similar results were also observed by Zeng et al. in the Cu-Sn foam electrode, that the total current decreased by almost half compared to that of pristine Cu foam [42]. Here, the LSV curves had different features. The overall current density did not show a great difference, even at −0.8 V vs. RHE when a big selectivity deviation is observed. HER is undoubtedly suppressed here, since the H_2_ current is reduced, but solely suppressed HER may not be enough to explain such an observation and it is speculated that the improvement of the intrinsic activity towards CO formation would be another reason.

**Figure 4:**
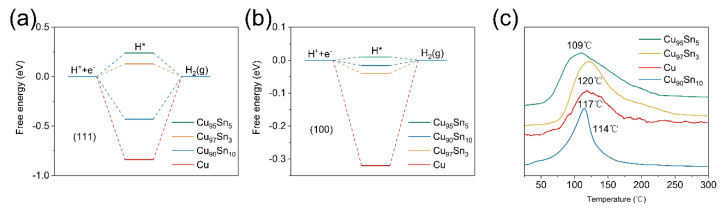
(**a**) Free energies of H* adsorbed on the (111) facet. (**b**) Free energies of H adsorbed on the (100) facet. (**c**) TPD-MS spectra of CO adsorbed on the Cu, Cu_97_Sn_5_, Cu_95_Sn_5_, Cu_90_Sn_10_ aerogels.

To prove this point, a calculation based on density function theory (DFT) was performed to see how the active site is altered between the Cu and Cu_x_Sn_y_ alloy aerogels, which makes the electrode highly selective toward CO [48,49,50,51,52]. The calculation aims to verify the suppressed HER on the Cu_x_Sn_y_ aerogels and focuses on a Cu cluster with 100 Cu atoms. An Sn atom can be placed on the face or inside of this cluster [29]. The effect of replacing a Cu atom with one Sn atom was first simulated to gain a reasonable configuration. Figure S6 shows the model of one Sn atom replacing one Cu atom on the (111) facet and in the inner structure, and the formation energy on the surface is −1.54 eV and for the inner structure is 0.40 eV, which indicates that the Sn atom preferentially replaces a Cu atom on the (111) plane. Then, the adsorption energy of key intermediates on the (100) and (111) facets of Cu is investigated to gain information on the possible effect of nearby Sn on the surface. In the case of H_2_ production, H* would be the relevant intermediate and the limiting step is suggested to be the formation of H* [41]. As shown in Figure 4a,b, Figures S7 and S8, H* can be easily adsorbed on surfaces (111) and (100) of Cu due to the more negative free energies of −0.87 eV and −0.32 eV, respectively. After Sn is introduced, such free energies become more positive—0.13 eV and −0.04 eV for Cu_97_Sn_3_ (111) and (100), 0.24 eV and 0.01 eV for Cu_95_Sn_5_ (111) and (100), and −0.43 eV and −0.016 eV for Cu_90_Sn_10_ (111) and (100), respectively. This indicates that an external energy barrier needs to be overcome to ensure effective H* adsorption on the Cu_x_Sn_y_ aerogel surfaces [24]. Therefore, the suppressed HER is evident, and the free energy of H* adsorption on the Cu_95_Sn_5_ aerogel is the most positive among these samples, which is in line with its lowest H_2_ partial current.

Besides, it is often proposed that the stepwise reactions for the reduction of CO2 to CO in an aqueous solution can be summarized as [19,53]:

CO_2_(g) + * + H^+^(aq) + e^−^ → COOH* (1)

COOH* + e^−^ + H^+^(aq) → CO* + H_2_O(l) (2)

and CO* → CO(g) + *(3)

In the third step, CO is released from the surface and is usually inhibited by strong CO binding on the catalyst, which can be considered one of the rate-determining steps [23]. In this case, the desorption capability of CO is important. The desorption behavior of CO on the Cu_x_Sn_y_ and Cu aerogels was then studied by TPD-MS (temperature-programmed desorption mass spectroscopy), which can give hints as to the binding strength of CO on the catalyst surface. Figure 4c shows the TPD profiles of CO on different metal aerogels. For all samples, a broad desorption peak is presented. The desorption peak temperature of CO for the Cu aerogel is located at 117 °C, while in the Cu_95_Sn_5_ aerogel, it is the lowest (109 °C). Thus, it can be concluded that alloying Sn in Cu aerogels leads to a decrease in the adsorption affinity of CO intermediates during CO_2_ reduction compared to monometallic Cu aerogels. The relatively weak binding to CO on CuxSny can accelerate CO production because CO can be easily released from the surface before it is further reduced to products, such as alcohols and hydrocarbons. With more Sn presented in the Cu_90_Sn_10_ aerogel, the desorption peak temperature of CO becomes higher to 114 °C. Therefore, the Cu_95_Sn_5_ aerogel shows the highest CO selectivity due to its promotion of CO production and inhibition of HER.

## 4. Conclusions

In conclusion, the Cu_x_Sn_y_ alloy aerogels were prepared by the simple co-reduction method. The synthesis is accomplished by the rapid reduction of the metal cations with the aid of NaBH_4_. The composition can be tuned by adjusting the precursor. The electrocatalytic reduction of CO_2_ was investigated on a series of Cu_x_Sn_y_ aerogels. The Cu_95_Sn_5_ aerogel electrode is a very efficient and stable electrocatalyst and the faradaic efficiency of CO production can reach 93% with a current density of 6.58 mA cm^−2^ at −0.9 V vs. RHE. The excellent catalytic performance of the Cu_95_Sn_5_ aerogel is mainly from the inhibition of HER and the promotion of CO_2_ conversion to CO simultaneously.

## Data Availability

Data is available upon reasonable request to the corresponding authors.

## Supplementary Materials

The following supporting information can be downloaded at: https://www.mdpi.com/article/10.3390/molecules28031033/s1, including additional characterization results of the samples.

## Sample Availability

Samples of the alloy aerogels samples are available from the authors.
