# Supplementary material for: Cu-Sn Aerogels for Electrochemical CO2 Reduction with High CO Selectivity
Source: Molecules. 2023 Jan 19;28(3):1033. doi: 10.3390/molecules28031033 (PMC9919718; doi:10.3390/molecules28031033)
Supplement: Supplementary file 1 [file molecules-28-01033-s001.zip › molecules-2117374-supplementary.pdf]

Cu-Sn Aerogels for Electrochemical CO_2 Reduction with High CO Selectivity

Yexin Pan [1], Muchen Wu [1], Ziran Ye [2,*], Haibin Tang [3], Zhanglian Hong [1] and Mingjia Zhi [4,*]

[1] State Key Laboratory of Silicon Material, School of Materials Science and Engineering, Zhejiang University, 38 Zheda Road, Hangzhou, 310027, China

[2] Department of Applied Physics, Zhejiang University of Technology, Hangzhou, 310014, China

[3] Key Laboratory of Materials Physics, and Anhui Key Laboratory of Nanomaterials and Nanotechnology, Hefei Institute of Solid State Physics, Chinese Academy of Sciences, Hefei, 230031, China

[4] Institute for Composites Science Innovation (InCSI), Zhejiang University, 38 Zheda Road, Hangzhou 310027, China

*Corresponding authors: E-mail: yeziran@zjut.edu.cn; Mingjia_zhi@zju.edu.cn

Table S1. Chemicals used in the synthesis of the hydrogels

Cu_xSn_y aerogels	$CuCl_2$ (mM)	$SnCl_4$ (mM)	Atomic Composition determined by ICP-AES
Cu	0.840	0	Cu 100%
$Cu_{97}Sn_3$	0.798	0.042	Cu:Sn=97:3
$Cu_{95}Sn_5$	0.756	0.084	Cu:Sn=95:5
$Cu_{90}Sn_{10}$	0.672	0.168	Cu:Sn=90:10
Sn	0	0.840	Sn 100%

Calculations of Faradaic efficiencies of gaseous products

The volume of the sample loop (V_0) for the gaseous product in our gas chromatograph is 1 cm^3 and the flow rate of the gas v = 20 cm^3/min. The time it takes to fill the sample loop is:

$$t = \frac{V_0}{v} = \frac{1\ cm^3}{20\ cm^3/min} = 0.05\ min = 3\ s \qquad (1)$$

According to the ideal gas law, under the ambient temperature of 25 °C, the amount of gas in each vial (V_0 = 1 cm^3) is:

$$n = \frac{P * V_0}{R * T} = \frac{1.013 * 10^5\ Pa * 1 * 10^{-6}\ m^3}{8.314\ J \cdot K^{-1} \cdot mol^{-1} * 298.150\ K} = 4.086 * 10^{-5}\ mol \qquad (2)$$

The number of electrons required to form 1 molecule of CO, CH_4, C_2H_4, or C_2H_6 is respectively 2, 8, 12, or 14. Take **Vial 3** and the calculation of **CO** as an example. The number of electrons (N_{CO}) needed to get x_0 ppm of CO is:

$$N_{CO} = x_0 * n * N_A * 2e$$

$$= 2514.639 * 10^{-6} * 4.086 * 10^{-5} mol * 6.02 * 10^{23} mol^{-1} * 2e$$

$$= 1.237 * 10^{17} e \qquad (3)$$

At the injection time to fill up vial 3, for example, the recorded current is I_0 = 7.08 mA (this data is obtained from the chronoamperogram). The total number of electrons (N_{total}) measured during this sampling period:

$$N_{total} = \frac{I_0 * t}{e} = \frac{7.08 * 10^{-3} A * 3s}{1.602 * 10^{-19} C/e} = 1.326 * 10^{17} e \qquad (4)$$

Hence, the faradic efficiency of CO is **(3)/(4)**:

$$FE = \frac{N_{CO}}{N_{total}} * 100\% = \frac{1.237 * 10^{17}}{1.326 * 10^{17}} = 93.28\% \qquad (5)$$

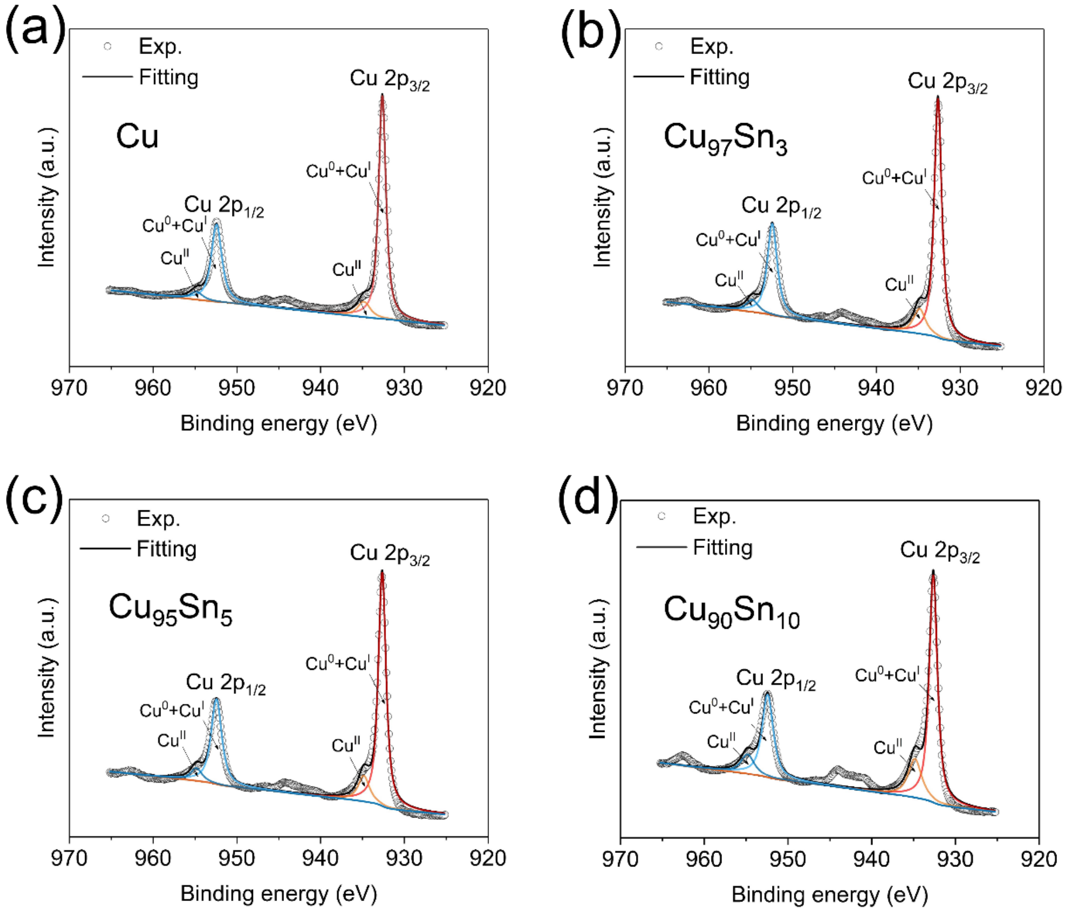

Figure S1. Cu 2p core level scan of the prepared aerogels: (a) Cu, (b) $Cu_{97}Sn_3$, (c) $Cu_{95}Sn_5$, and (d) $Cu_{90}Sn_{10}$.

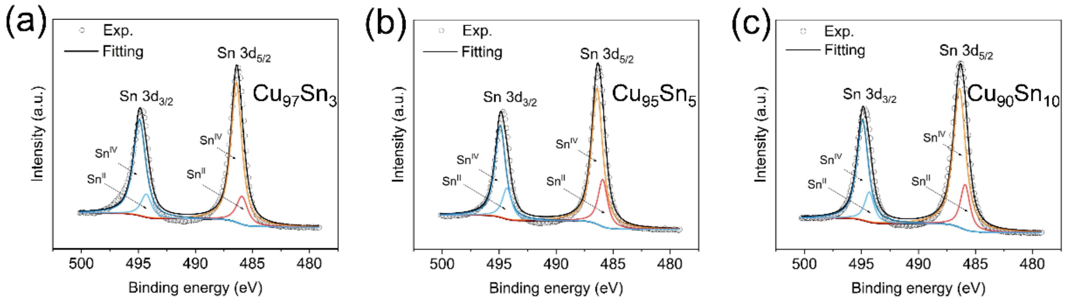

Figure S2. Sn 3d core level scan of prepared aerogels: (a) $Cu_{97}Sn_3$, (b) $Cu_{95}Sn_5$, and (c) $Cu_{90}Sn_{10}$.

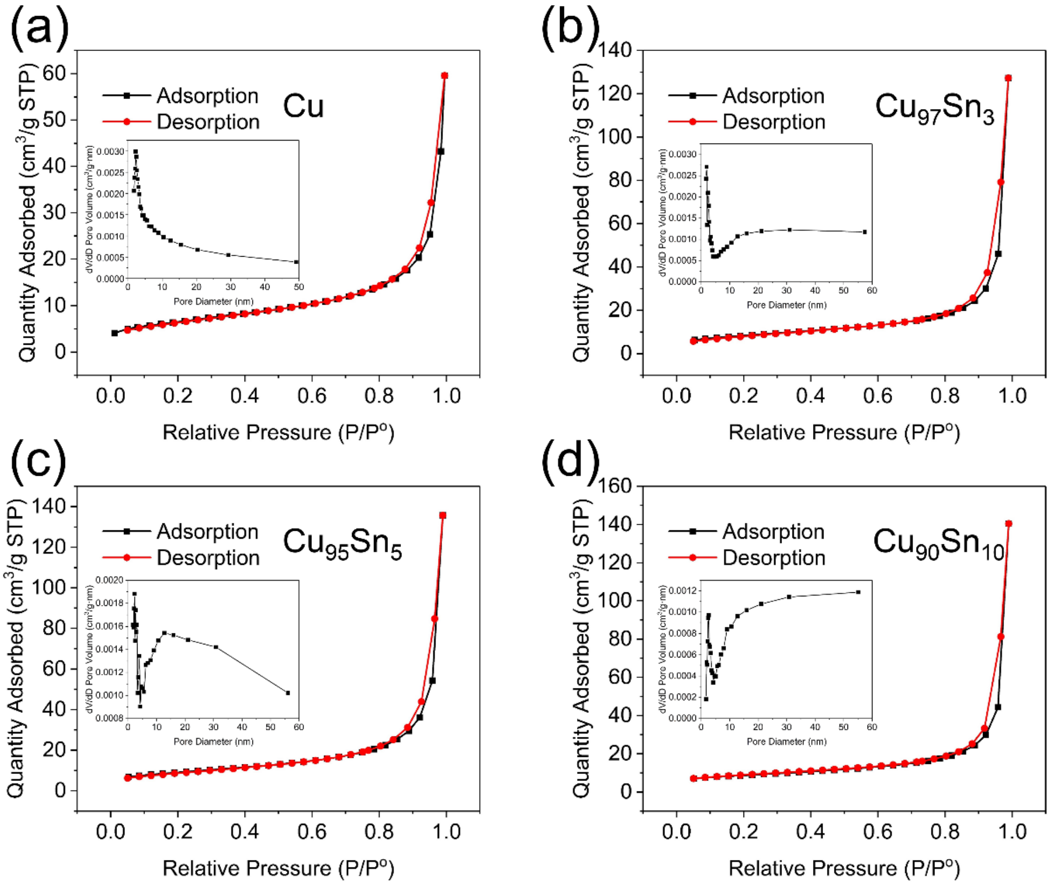

Figure S3. N_2 physisorption isotherms for the as-prepared aerogels: (a) Cu, (b) $Cu_{97}Sn_3$, (c) $Cu_{95}Sn_5$, and (d) $Cu_{90}Sn_{10}$.

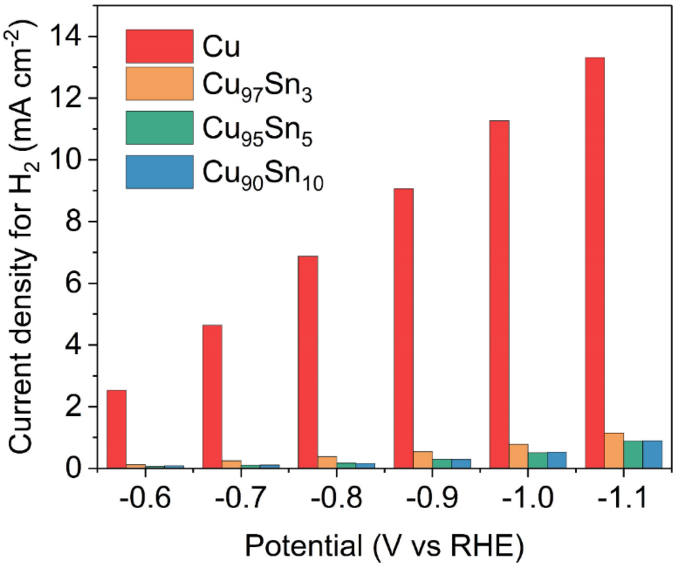

Figure S4. H_2 partial current density at different potentials for Cu, $Cu_{97}Sn_3$, $Cu_{95}Sn_5$, $Cu_{90}Sn_{10}$ aerogels.

Table S2 The comparison of CO FE, total current density, and CO current density of different Cu based electrodes at different potentials

Sample	FE_{CO} (%)	Total current density (mA cm^{-2})	CO current density (mA cm^{-2})
-0.6 V *vs* RHE			
Cu-In bimetal[1]	90	1.1	1.0
Cu-Sn foam[2]	90	1.3	1.2
Cu-Sn bimetal[3]	92	1.3	1.2
$Cu_{95}Sn_5$ (this work)	87	1.8	1.6
-0.7 V *vs* RHE			
Cu-In bimetal[1]	90	1.7	1.5
Cu-Ag bimetal[4]	46	1.2	0.6
Cu-Sn bimetal[3]	90	2.1	1.9
$Cu_{95}Sn_5$ (this work)	90	3.2	2.8
-0.8 V *vs* RHE			
Cu-Ag bimetal[4]	74	3.4	2.5
Cu-Sn foam[2]	91	4.9	4.5
Cu-Sn bimetal[3]	92	3.3	3.0
$Cu_{95}Sn_5$ (this work)	92	5.0	4.6
-0.9 V *vs* RHE			
Cu-Ag bimetal[4]	90	5.9	5.3
Cu-Sn foam[2]	91	6.8	6.2
$Cu_{95}Sn_5$ (this work)	93	7.1	6.6

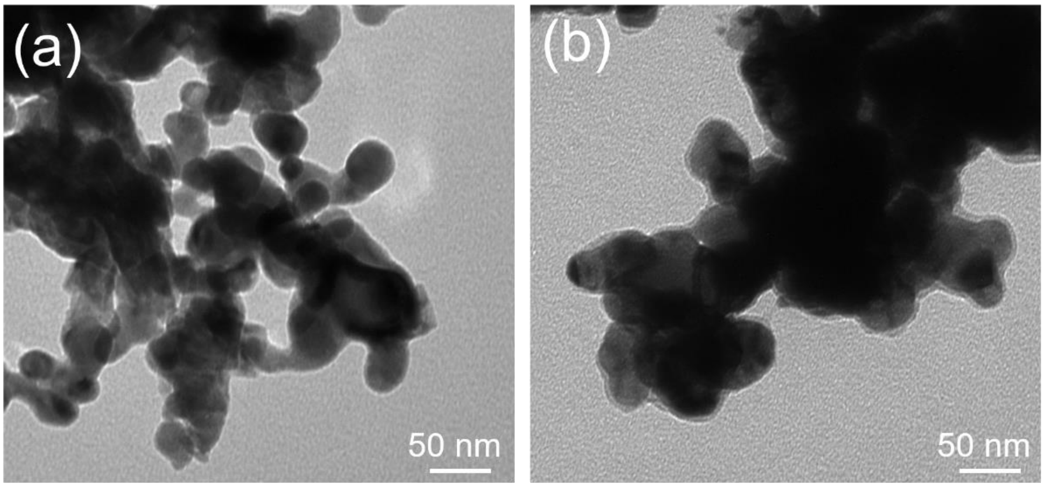

Figure S5. TEM images of $Cu_{95}Sn_5$ aerogel electrode: (a) before electrolysis and (b) after 20 hours of electrolysis.

DFT computations

All calculations were carried out using density functional theory as implemented in the CP2K package.[5] The generalized-gradient approximation (GGA), as parameterized by Perdew, Burke, and Ernzerhof (PBE) was utilized to compute the exchange-correlation energy.[6,7] Dispersion interactions were considered in calculations by using an empirical correction scheme developed by PBE (D3).[8] All atoms were described with the DZVP-MOLOPT-GTH pseudopotentials.[9]

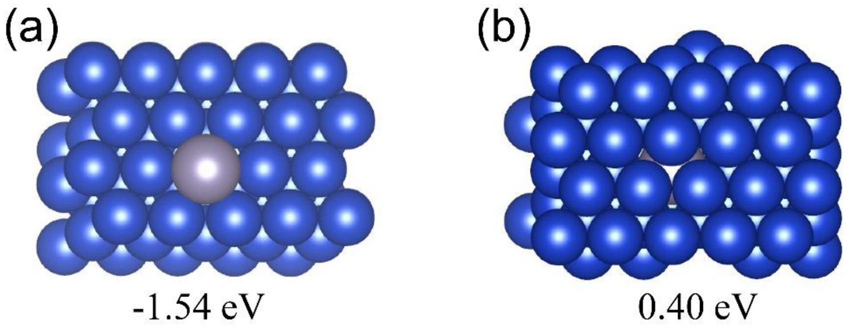

Figure S6. Possible positions of an Sn atom replacing a Cu atom on a Cu (100) structure to form a $Cu_{99}Sn_1$ structure: (a) face, (b) inner.

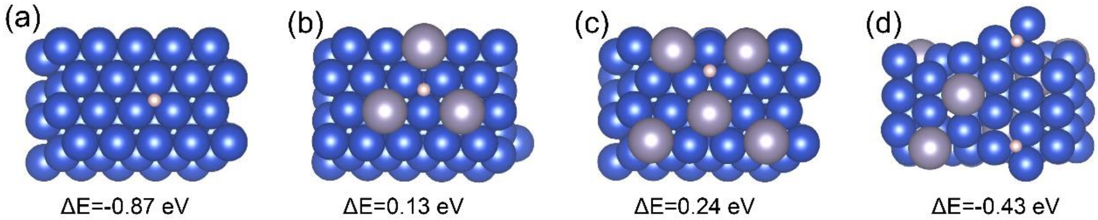

Figure S7. Free energies of H adsorbed on the (a) Cu, (b) $Cu_{97}Sn_3$, (c) $Cu_{95}Sn_5$, (d) $Cu_{90}Sn_{10}$ (111) facet.

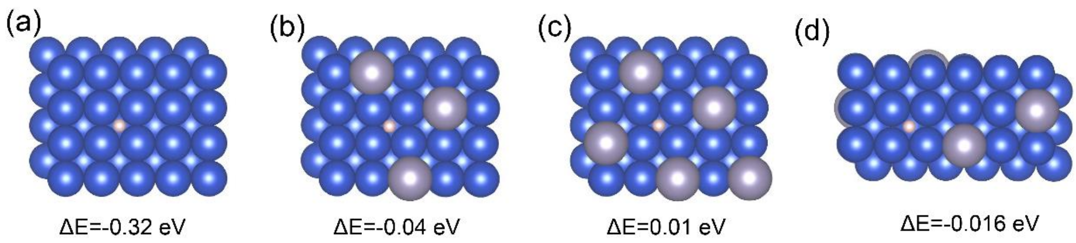

Figure S8. Free energies of H adsorbed on the (a) Cu, (b) $Cu_{97}Sn_3$, (c) $Cu_{95}Sn_5$, (d) $Cu_{90}Sn_{10}$ (100) facet.

References

1. Rasul S, Anjum DH, Jedidi A, Minenkov Y, Cavallo L, Takanabe K (2015) A Highly Selective Copper-Indium Bimetallic Electrocatalyst for the Electrochemical Reduction of Aqueous CO_2 to CO. Angewandte Chemie-International Edition 54 (7):2146-2150. doi:10.1002/anie.201410233

2. Zeng J, Bejtka K, Ju W, Castellino M, Chiodoni A, Sacco A, Farkhondehfal MA, Hernandez S, Rentsch D, Battaglia C, Pirri CF (2018) Advanced Cu-Sn foam for selectively converting CO_2 to CO in aqueous solution. Applied Catalysis B-Environmental 236:475-482. doi:10.1016/j.apcatb.2018.05.056

3. Sarfraz S, Garcia-Esparza AT, Jedidi A, Cavallo L, Takanabe K (2016) Cu-Sn Bimetallic Catalyst for Selective Aqueous Electroreduction of CO_2 to CO. Acs Catalysis 6 (5):2842-2851. doi:10.1021/acscatal.6b00269

4. Wang W, Gong S, Liu J, Ge Y, Wang J, Lv X (2021) Ag-Cu aerogel for electrochemical CO2 conversion to CO. Journal of Colloid and Interface Science 595:159-167. doi:10.1016/j.jcis.2021.03.120

5. Kuehne TD, Iannuzzi M, Del Ben M, Rybkin VV, Seewald P, Stein F, Laino T, Khaliullin RZ, Schutt O, Schiffmann F, Golze D, Wilhelm J, Chulkov S, Bani-Hashemian MH, Weber V, Borstnik U, Taillefumier M, Jakobovits AS, Lazzaro A, Pabst H, Mueller T, Schade R, Guidon M, Andermatt S, Holmberg N, Schenter GK, Hehn A, Bussy A, Belleflamme F, Tabacchi G, Gloss A, Lass M, Bethune I, Mundy CJ, Plessl C, Watkins M, VandeVondele J, Krack M, Hutter J (2020) CP2K: An electronic structure and molecular dynamics software package - Quickstep: Efficient and accurate electronic structure calculations. Journal of Chemical Physics 152 (19). doi:10.1063/5.0007045

6. Perdew JP (1986) DENSITY-FUNCTIONAL APPROXIMATION FOR THE CORRELATION-ENERGY OF THE INHOMOGENEOUS ELECTRON-GAS. Physical Review B 33 (12):8822-8824. doi:10.1103/PhysRevB.33.8822

7. Perdew JP, Burke K, Ernzerhof M (1996) Generalized gradient approximation made simple. Physical Review Letters 77 (18):3865-3868. doi:10.1103/PhysRevLett.77.3865

8. Grimme S, Antony J, Ehrlich S, Krieg H (2010) A consistent and accurate ab initio parametrization of density functional dispersion correction (DFT-D) for the 94 elements H-Pu. Journal of Chemical Physics 132 (15). doi:10.1063/1.3382344

9. Goedecker S, Teter M, Hutter J (1996) Separable dual-space Gaussian pseudopotentials. Physical Review B 54 (3):1703-1710. doi:10.1103/PhysRevB.54.1703
